# High androgen concentrations in follicular fluid of polycystic ovary syndrome women

**DOI:** 10.1186/s12958-022-00959-6

**Published:** 2022-06-14

**Authors:** Alice Bongrani, Ingrid Plotton, Namya Mellouk, Christelle Ramé, Fabrice Guerif, Pascal Froment, Joëlle Dupont

**Affiliations:** 1 UMR 85 Physiology of Reproduction and Behaviour, National Research Institute for Agriculture, Food and Environment (INRAE) Centre Val de Loire, 37380 Nouzilly, France; 2 Molecular Endocrinology and Rare Diseases, University Hospital, Claude Bernard Lyon 1 University, 69677 Bron, France; 3 UMR 85 Physiology of Reproduction and Behaviour, National Research Institute for Agriculture, Food and Environment (INRAE) Ile de France, 78352 Jouy-en-Josas, France; 4 Reproductive Medicine and Biology Department, University Hospital of Tours, 37000 Tours, France

**Keywords:** Androgens, Polycystic ovary syndrome (PCOS), Theca cells, Follicular fluid, Adipokines

## Abstract

**Background:**

According to current definitions of Polycystic Ovary Syndrome (PCOS), hyperandrogenism is considered as a key element in the pathogenesis of this common endocrinopathy. However, until now, studies about ovarian androgen profile in women are very rare. Our aim was then to characterise the expression profile of the androgens in follicular fluid of 30 PCOS patients, and compare it to those of 47 Control women and 29 women with only polycystic ovary morphology on ultrasounds (ECHO group).

**Methods:**

A retrospective, single-centre cohort study was performed. The intrafollicular concentrations of the key androgens were assessed and correlated with the intrafollicular levels of some adipokines of interest. Androgens were quantified by mass spectrophotometry combined with ultra-high-performance liquid chromatography, while adipokine concentrations were measured by ELISA assays.

**Results:**

In PCOS patients, the intrafollicular concentrations of the androgens synthesised by ovarian theca cells, i.e., 17OH-pregnenolone, dehydroepiandrosterone, Δ4-androstenedione and testosterone, were significantly higher than those of the androgens of adrenal origin, and positively correlated with the main PCOS clinical and biological features, as well as with the adipokines mostly expressed in the follicular fluid of PCOS patients, i.e. resistin, omentin, chemerin and apelin. Conversely, Control women showed the highest levels of 17OH-progesterone, deoxycorticosterone and 11-deoxycortisol. Confirming these results, apelin levels were negatively associated with pregnenolone and deoxycorticosterone concentrations, while visfatin levels, which were higher in the Control group, negatively correlated with the Δ4-androstenedione and testosterone ones.

**Conclusions:**

PCOS is characterised by a selective increase in the intrafollicular levels of the androgens synthesised by theca cells, strengthening the hypothesis that ovarian hyperandrogenism plays a central role in its pathogenesis. Further, the significant correlation between the intrafollicular concentrations of the androgens and most of the adipokines of interest, including apelin, chemerin, resistin and omentin, confirms the existence of a close relationship between these two hormonal systems, which appear deeply involved in ovarian physiology and PCOS physiopathology.

## Background

Although according to current recommendations its presence is not mandatory for the diagnosis [1], hyperandrogenism is considered as a key element in the pathogenesis of Polycystic Ovary Syndrome (PCOS). This position is especially supported by the Androgen Excess and PCOS Society, that has strongly opposed the ESHRE/ASRM definition of PCOS since its introduction in 2003 [2], considering androgen excess as an essential diagnostic criterion [3]. Indeed, over the last decades, several authors highlighted in PCOS women the existence of a hyperandrogenic state mostly resulting from an intrinsic alteration of ovarian theca cells (TC) [4, 5]. These data have been further corroborated by the results obtained in vitro and in animal models, underlining the central role of ovarian hyperandrogenism in the development of PCOS cardinal features [6–8].

As known, PCOS is associated with a significant metabolic impact [9, 10] partially linked to adipocyte dysfunction. Indeed, women with PCOS secrete unbalanced levels of plasma adipokines [11, 12], characterised by higher concentrations of leptin [13] and alterations in plasma and ovarian adiponectin, chemerin, resistin, visfatin, apelin and omentin, that might be directly or indirectly involved in PCOS pathogenesis [11, 14]. Hyperandrogenism itself drives dysfunctional adipocyte secretion of potentially harmful adipokines. Indeed, mice knocked down for adipocyte androgen receptor had alterations in adipokine levels, impaired insulin sensitivity and poor glucose tolerance not associated with obesity [15]. Even in non-obese PCOS patients, androgen hypersecretion and androgen receptor dysfunction seem to underlie the changes in adipokine levels [16] suggesting a tight interaction between these two hormonal systems.

Despite the central role of hyperandrogenism in PCOS, until now, to our knowledge, androgen profile in women has been studied almost exclusively in blood plasma, and data about ovarian androgen synthesis is yet very rare [17]. Thus, we characterised the expression profile of the key androgens in the follicular fluid (FF) of a cohort of PCOS patients, and decided to compare it to those of Control women and women presenting only polycystic ovary morphology (PCOM) on ultrasounds. Indeed, this condition, that we named ECHO, is considered by several authors as a PCOS-like phenotype characterised by a mild dysfunction in ovarian steroidogenesis able to stimulate the early phases of folliculogenesis but not yet sufficient to affect the ovulatory process [5, 18]. Then, we correlated our results with the follicular concentrations of some adipokines of interest, namely adiponectin, chemerin, resistin, visfatin, omentin, apelin and vaspin, and analysed the possible relationships existing between these two hormonal systems in order to better define their role in ovarian physiology and especially in PCOS pathogenesis.

## Methods

### Study population

The study was carried out in accordance with the Declaration of Helsinki principles and free informed consent was obtained from all participants. Study protocol was previously approved by the Ethics Committee of the University Hospital of Tours as part of the “PREVADI” and “HAPOERTI” projects (authorisation number 2016_075).

The study population included all the women undergoing an in vitro fertilisation (IVF) procedure at the Reproductive Medicine and Biology Department of Tours University Hospital between 2011 and 2018 for whom we had all the samples and data of interest. Infertility causes included ovulatory insufficiency, diminished ovarian reserve, tubal diseases, endometriosis, male sterility or a combination of male and female causes. Women suffering from hyperprolactinemia and/or thyroid diseases were excluded. The ovarian stimulation and IVF protocols used [19] were the same for all included subjects. Clinical and biological information was retrospectively collected from the “INFO-FIV” and “Dossier Patient Partagé” databases of the University Hospital of Tours. Anthropometric parameters and clinical signs of hyperandrogenism, i.e. acne, alopecia and hirsutism, were recorded during the physical examination and a transvaginal pelvic ultrasound determining the antral follicle count was performed by an experienced sonographer during the early follicular phase. Venous blood samples were obtained between days 3 and 5 of the cycle for AMH, FSH, LH, and oestradiol dosages, while plasma testosterone was mainly assessed in the presence of elements supporting PCOS diagnosis. Notwithstanding, the presence of clinical and/or biological signs of hyperandrogenism was verified and excluded prior to inclusion for all the women included in the Control and ECHO groups.

A total of 106 subjects, aged between 21 and 42 years (31.6 ± 4.7) were allocated to 3 groups: PCOS group (*n* = 30), ECHO group, defined by the presence of PCOM without any other criteria necessary for PCOS diagnosis (*n* = 29), and Control group, including women with regular cycles and a follicle count < 12 per ovary (*n* = 47). According to the diagnostic criteria of the 2003 Rotterdam Consensus Conference [2], all PCOS patients presented with oligo-anovulation and PCOM, possibly associated with clinical and/or biological signs of hyperandrogenism. Each group was composed almost equally by normal-weight (Body Mass Index, BMI 18–24,9 kg/m^2^) and overweight/obese (BMI ≥ 25 kg/m^2^) subjects.

### Biological sampling

#### Blood analysis

Biological testing of venous blood samples was carried out in biomedical laboratories convenient to subjects’ home, and the data of interest, especially hormone dosage values, was retrospectively collected from medical records. However, as regards plasma testosterone, to make the data more homogeneous, we repeated the analyses in the blood samples available to our laboratory using an Immulite® 2500 Immunoassay analyser (Siemens, Munich, Germany).

#### Follicular fluid (FF)

FF was obtained during oocyte retrieval as part of the IVF procedure. Punction fluid was collected and centrifuged at 400 g for 10 minutes to separate the cellular remnants from the supernatant FF, which was then frozen at − 80 °C for subsequent analysis.

### Hormone assays

#### Androgen quantification

Androgens were quantified by mass spectrophotometry combined with ultra-high-performance liquid chromatography as described by Meunier et al. [20]. This is a newly developed technique that allows the simultaneous assay of several molecules with higher sensitivity and specificity than immunoassays, thus permitting the use of smaller sample volumes [21].

#### Adipokine assays

Adipokine concentration in FF was measured by ELISA assay. An R&D System kit (Abingdon, United Kingdom) was used for adiponectin, chemerin, resistin, visfatin, omentin and apelin (intra- and inter-assay coefficients of variation < 6% and ≤ 8%, respectively), whereas for the vaspin assay we employed a MyBioSource kit (San Diego, USA) with a sensitivity of 0.05 ng/L and intra- and inter-assay coefficients of variation < 12 and < 8%, respectively.

### Statistical analysis

Comparisons between groups were analysed by two-way ANOVA followed by Bonferroni post-hoc tests. Given the finding of a statistically significant interaction between weight and allocation group (Control, ECHO or PCOS), the analyses concerning BMI and follicle count were carried out separately for normal-weight and overweight/obese subjects (Table 1). Simple linear regression was used as appropriate. Results, expressed as mean ± standard deviation, were not adjusted for age or any other factor. Statistical analysis was performed using GraphPad Prism software (version 9.1.0, San Diego, CA, USA) and *p* < 0.05 was considered the statistical threshold for declaring significance.

**Table 1:** Clinical-biological features and in vitro fertilisation procedure outcomes of study women

	**Controls**		**ECHO**		**PCOS**		**BMI Effect**	**Condition Effect**
	**NW**	**Obese**	**NW**	**Obese**	**NW**	**Obese**		
**BMI **(kg/m^2^)	21.2 ± 2.0 **(***n* **= **26**)**	**31.5 ± 4.1**^**#**^ (*n* = 21)	21.6 ± 1.89 (*n* = 15)	**29.4 ± 3.0** (*n* = 14)	21.05 ± 1.8 (*n* = 13)	**34.06 ± 2.3**^**°**^ (*n* = 13)	***p*** **< 0.001**	***p*** **< 0.05**
**Follicle Count** (n)	16.3 ± 5.0 (*n* = 23)	11.1 ± 4.3 (*n* = 20)	**35.1 ± 13.0**^**∞**^ (*n* = 15)	**33.9 ± 4.5**^•^ (*n* = 14)	**39.1 ± 14.7**^**∞**^ (*n* = 17)	**33.0 ± 8.4**^•^ (*n* = 7)	***p*** **< 0.05**	***p*** **< 0.0001**
**Cycles Duration** (d)	28.3 ± 2.8 (*n* = 42)		30.5 ± 1.8 (*n* = 28)		79.1 ± 62.7^***/^^^^ (*n* = 25)		NS	***p*** **< 0.0001**
**Testosterone** (μg/L)	0.40 ± 0.20 (*n* = 23)		0.35 ± 0.12 (*n* = 18)		**0.61 ± 0.48**^**^**^ (*n* = 25)		NS	***p*** **< 0.05**
**AMH** (ng/mL)	2.68 ± 1.74 (*n* = 47)		6.06 ± 2.38 (*n* = 29)		11.10 ± 11.66^***/^^^ (*n* = 30)		NS	***p*** **< 0.0001**
**FSH** (UI/L)	6.44 ± 2.33 (*n* = 46)		6.13 ± 1.97 (*n* = 29)		5.75 ± 1.77 (*n* = 29)		NS	NS
**LH** (UI/L)	4.38 ± 1.65 (*n* = 46)		5.53 ± 2.65 (*n* = 28)		6.12 ± 2.96^**^ (*n* = 29)		NS	***p*** **< 0.01**
**Oestradiol** (ng/L)	46.87 ± 28.35 (*n* = 46)		41.56 ± 30.32 (*n* = 28)		37.03 ± 9.92 (*n* = 29)		NS	NS
**Matures Oocytes** (n)	6.5 ± 3.5 (*n* = 46)		10.3 ± 6.1^**^ **(*n*** **= 29)**		8.3 ± 4.8 (*n* = 29)		NS	***p*** **< 0.01**
**Embryos** (n)	3.9 ± 3.0 (*n* = 46)		7.1 ± 4.4^***^ **(*n*** **= 29)**		5.4 ± 3.3 (*n* = 29)		NS	***p*** **< 0.01**

*Note*: The values are expressed as mean ± standard deviation. *ECHO* women presenting ≥12 follicles per ovary without dysovulation and/or hyperandrogenism; *PCOS* Polycystic Ovary Syndrome; *NW* Normal-Weight; *BMI* Body Mass Index; *AMH* Anti-Müllerian Hormone; *FSH* Follicle-Stimulating Hormone; *LH* Luteinising Hormone

^#^*p* < 0.05 versus obese ECHO; ^°^*p* < 0.0001 versus obese ECHO; ^∞^*p* < 0.0001 versus normal-weight Controls; ^•^*p* < 0.0001 versus obese Controls; * *p* < 0.05, ** *p* < 0.01, *** *p* < 0.001 versus Controls; ^ *p* < 0.05, ^^ *p* < 0.01, ^^^ *p* < 0.001 versus ECHO

## Results

### Study population description

The anthropometric, clinical and biological features of study population, as well as IVF procedure outcomes, are reported in Table 1. According to the patient assignment criteria, PCOS group was characterised by 2.5 times longer cycles duration, and follicle count was significantly higher for both PCOS and ECHO women. Similarly, in agreement with literature [22], PCOS patients had higher plasma testosterone, AMH and LH levels, while FSH and oestradiol blood concentrations did not differ among groups. Based on the oocytes and embryos counts, as we already pointed out in our previous studies [23, 24], the ECHO group seemed to be characterised by the best IVF success rates, although statistically significant differences were limited to comparisons with the Control group.

### The ovary at the origin of the hyperandrogenism characterising PCOS

**Fig. 1:**
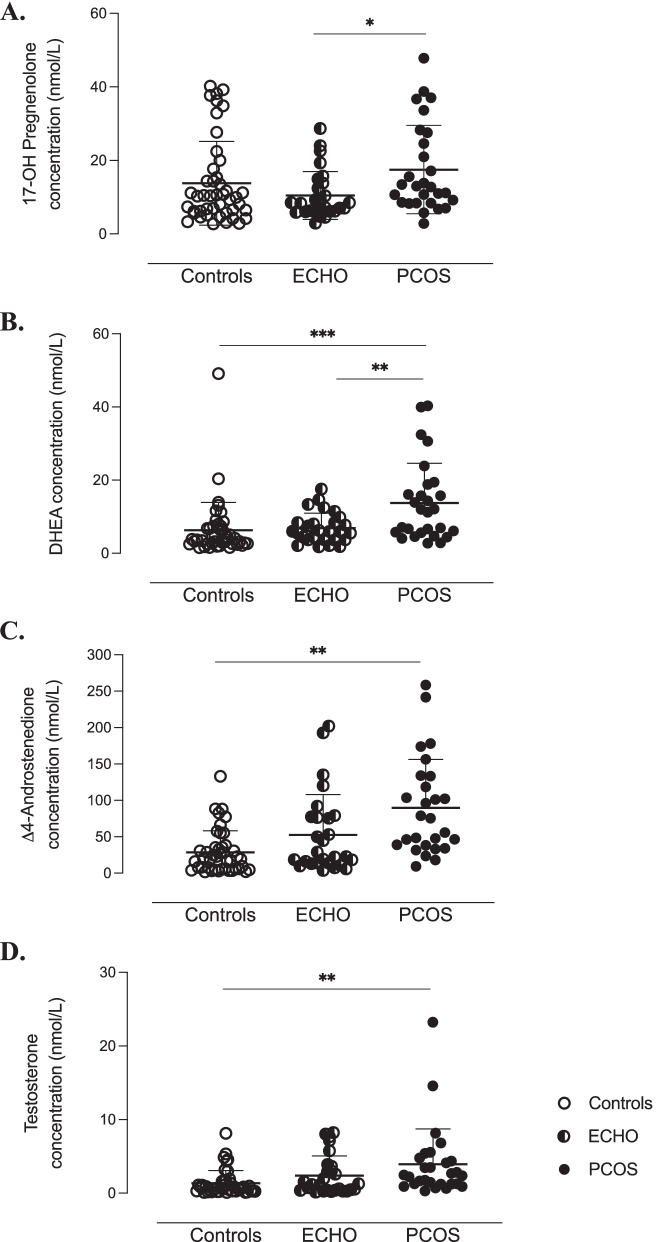
Ovary-synthetised androgens in follicular fluid of study women. Follicular concentrations of 17OH-pregnenolone (**A**), dehydroepiandrosterone (DHEA) (**B**), ∆4-androstenedione (**C**) and testosterone (**D**) quantified by mass spectrophotometry combined with ultra-high performance liquid chromatography (LC-MS/MS). ^***^p < 0.001, ^**^p < 0.01, ^*^p < 0.05 (two-way ANOVA followed by Bonferroni post-hoc tests)

**Fig. 2:**
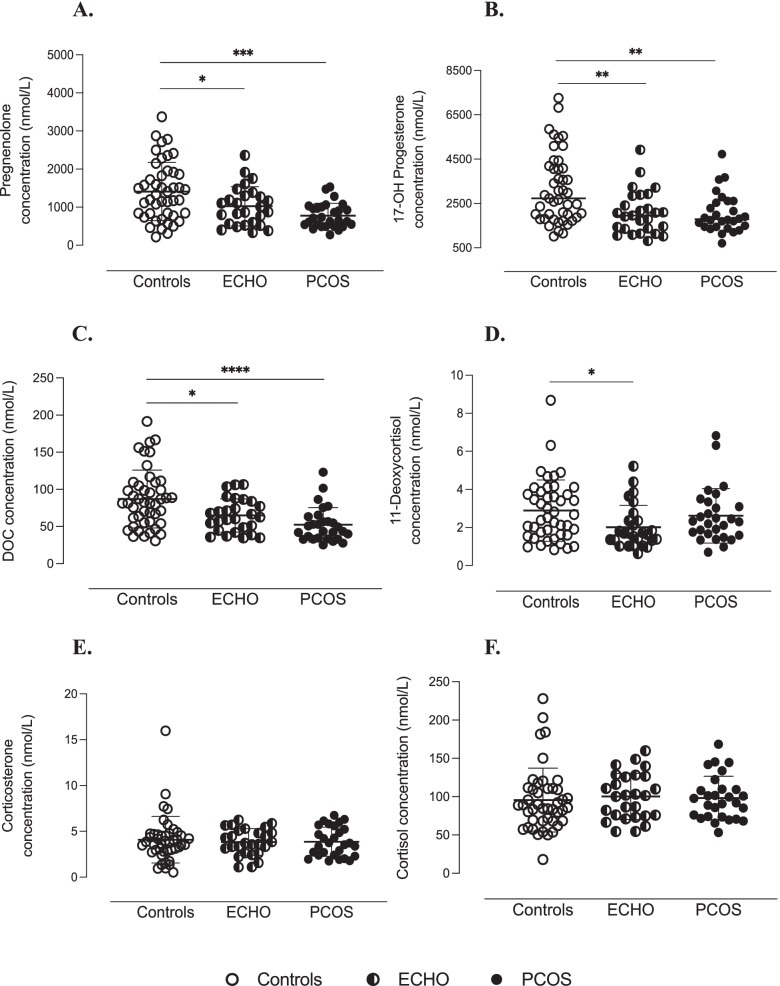
Adrenal-originated androgens in follicular fluid of study women. Follicular concentrations of pregnenolone (**A**), 17OH-progesterone (**B**), deoxycorticosterone (DOC) (**C**), 11-deoxycortisol (**D**), corticosterone (**E**) and cortisol (**F**) quantified by mass spectrophotometry combined with ultra-high performance liquid chromatography (LC-MS/MS). ^****^p < 0.0001, ^***^ p < 0.001, ^**^ p < 0.01, ^*^p < 0.05 (two-way ANOVA followed by Bonferroni post-hoc tests)

As reported in Figs. 1 and 2, in PCOS patients, follicular concentrations of the androgens synthesised by TC, i.e., 17OH-pregnenolone, dehydroepiandrosterone (DHEA), Δ4-androstenedione and testosterone, were significantly higher than those of adrenal origin. The opposite situation was observed in the Control group. Indeed, Control women showed the highest levels of 17OH-progesterone, deoxycorticosterone (DOC) and 11-deoxycortisol, as well as the lowest levels of 17OH-pregnenolone, DHEA, Δ4-androstenedione and testosterone. Interestingly, in the ECHO group, follicular concentrations of most ovarian-derived androgens were intermediate between PCOS patients and Controls. No significant variation was observed in corticosterone and cortisol levels (Fig. 2).

### Hyperandrogenism is tightly associated to the major clinical and biological features of PCOS

**Fig. 3:**
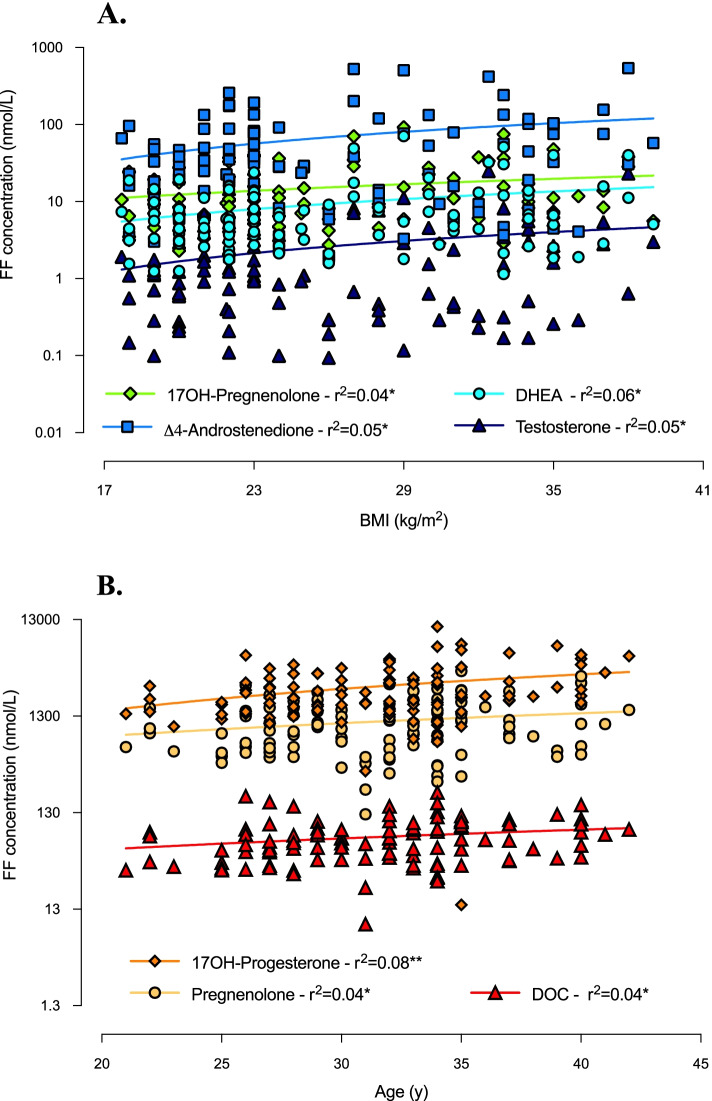
Correlations between androgen concentrations in follicular fluid and anthropometric features of study women. Correlations of androgen levels in follicular fluid (FF) with BMI (**A**) and age (**B**) (simple linear regression). ^*^p ≤ 0.05 and ^**^p < 0.01

**Fig. 4:**
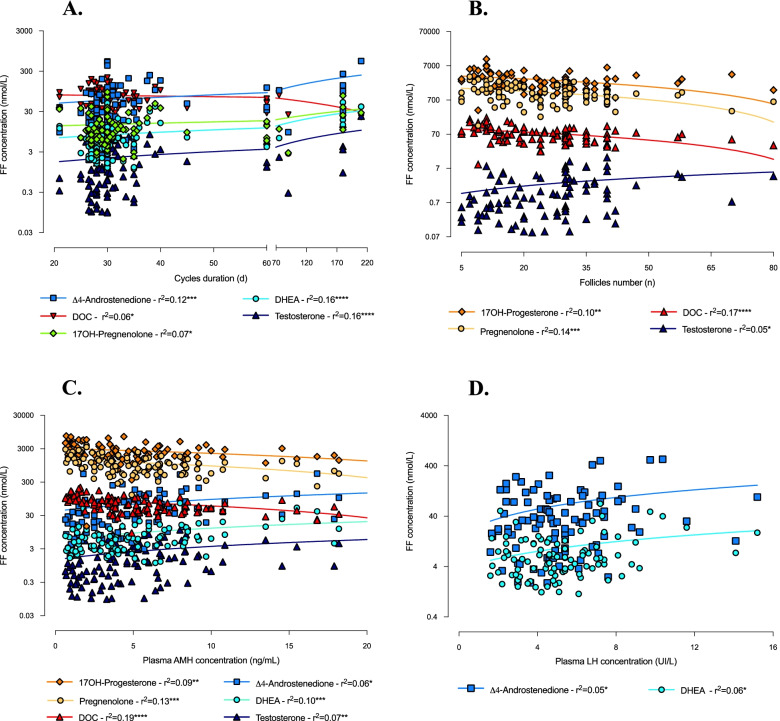
Correlations between androgen follicular concentrations and clinical-biological parameters of study women. Correlations of androgen levels in follicular fluid (FF) with cycles duration (**A**), follicles count (**B**), plasma AMH (**C**) and plasma LH (**D**) concentrations (simple linear regression). ^*^p ≤ 0.05, ^**^p < 0.01, ^***^p < 0.001 and ^****^p < 0.0001

As illustrated in Figs. 3 and 4, follicular concentrations of 17OH-pregnenolone, DHEA, Δ4-androstenedione and testosterone significantly positively correlated with BMI and cycles duration. Additionally, DHEA, Δ4-androstenedione and testosterone levels were significantly positively associated with plasma concentrations of AMH and LH (for LH, however, a significant *p*-value was obtained only in associations with DHEA and Δ4-androstenedione). As regards testosterone concentration, we also found a significant positive correlation with ovarian follicle count, which, by contrast, was negatively associated with the levels of the adrenal-originated androgens (Fig. 4). The latter, i.e.*,* 17OH-progesterone, pregnenolone and DOC, also correlated negatively with plasma AMH concentration (Fig. 4) and positively with age (Fig. 3). No significant association was found regarding corticosterone and cortisol levels.

### Relationships between androgens and adipokines in FF

**Fig. 5:**
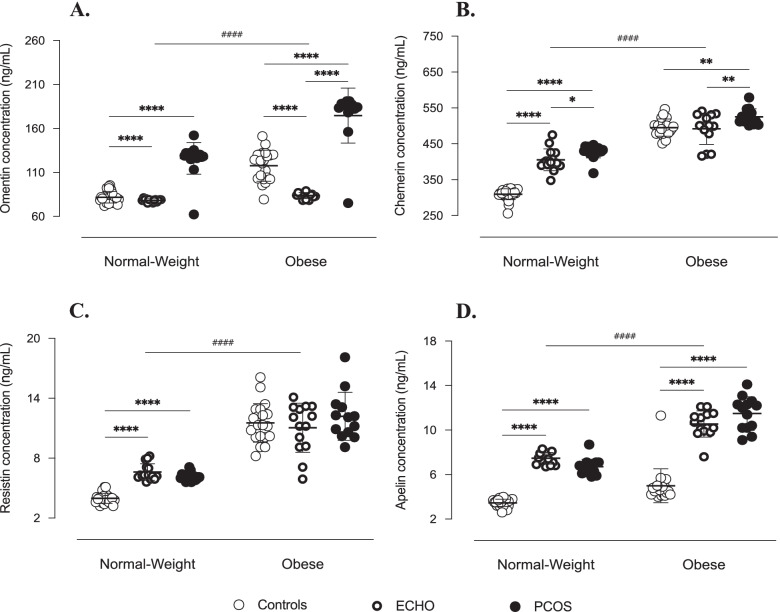
Adipokines in follicular fluid of study women. Omentin (**A**), chemerin (**B**), resistin (**C**) and apelin (**D**) concentrations measured by ELISA assay in follicular fluid. Data are shown as individual values with means as horizontal bars. Groups were compared by two-way ANOVA followed by Bonferroni post-hoc tests. As interactions between BMI and pathological condition were statistically significant (*p* < 0.001), normal-weight and obese groups were analysed separately. ^****/####^*p* < 0.0001, ^**^p < 0.01, ^*^p < 0.05

**Fig. 6:**
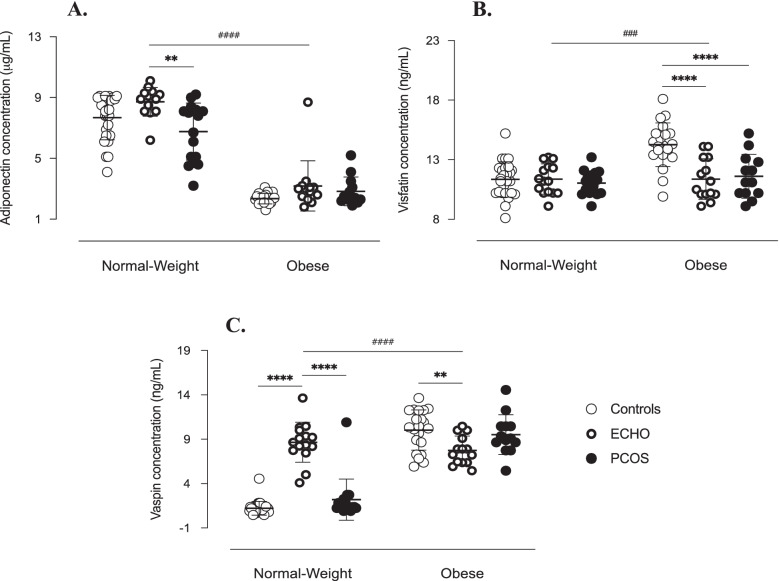
Adipokines in follicular fluid of study women. Adiponectin (**A**), visfatin (**B**) and vaspin (**C**) concentrations measured by ELISA assay in follicular fluid. Data are shown as individual values with means as horizontal bars. Groups were compared by two-way ANOVA followed by Bonferroni post-hoc tests. As interactions between BMI and pathological condition were statistically significant (*p* < 0.001), normal-weight and obese groups were analysed separately. ^****/####^*p* < 0.0001, ^###^*p* < 0.001, ^**^p < 0.01

The data about adipokine levels in FF largely reflected those described in our previous papers [23, 24]. Follicular concentrations of all adipokines of interest were mainly and significantly influenced by BMI. Indeed, while normal-weight women had the highest levels of adiponectin, the concentrations of visfatin, resistin, chemerin, omentin, apelin and vaspin were largely predominant in obese subjects (Figs. 5 and 6).

Concerning the pathological status, however, the results were less unified. PCOS patients had higher concentrations of chemerin, omentin, apelin and resistin (Fig. 5). The ECHO group also showed significantly higher levels of apelin and, although limited to normal-weight subgroup, of resistin, adiponectin and vaspin (Figs. 5 and 6). In turn, follicular concentration of visfatin was lower in obese ECHO and PCOS subjects than in BMI-matched Controls (Fig. 6).

**Fig. 7:**
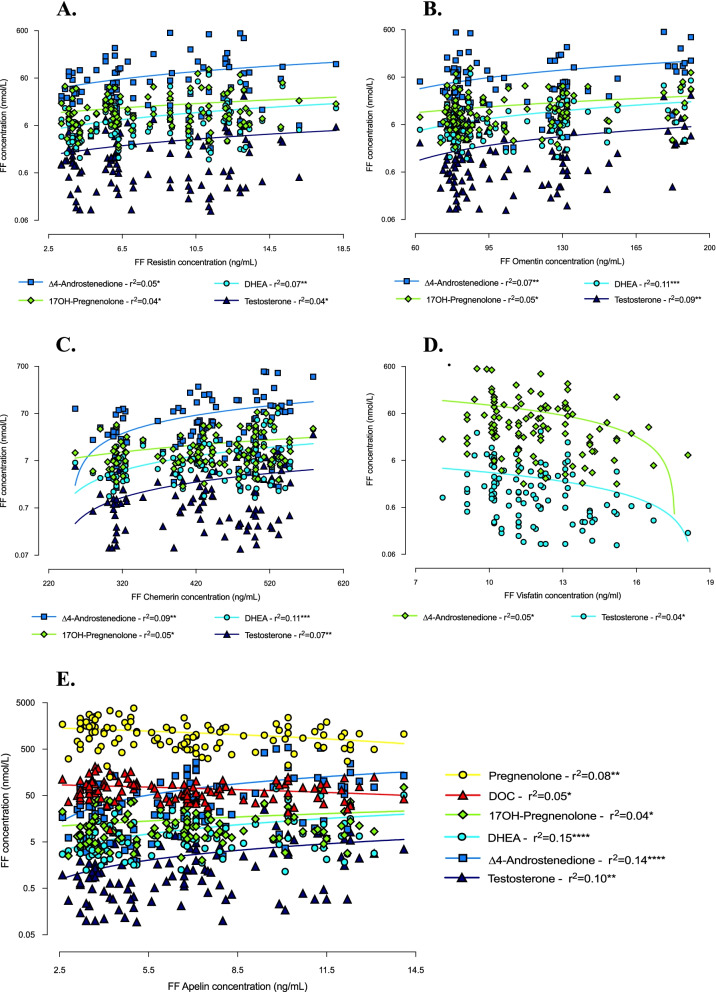
Correlations between androgen and adipocytokine concentrations in follicular fluid of study women. Correlations between androgen levels in follicular fluid and resistin (**A**), omentin (**B**), chemerin (**C**), visfatin (**D**) and apelin (**E**) follicular concentrations (simple linear regression). ^*^p ≤ 0.05, ^**^p < 0.01, ^***^p < 0.001 and ^****^p < 0.0001. DHEA = dehydroepiandrosterone; DOC = deoxycorticosterone

Interestingly, the concentrations of the most expressed androgens and adipokines in FF of PCOS patients (17OH-pregnenolone, DHEA, Δ4-androstenedione and testosterone, and resistin, omentin, chemerin and apelin, respectively) positively correlated each other (Fig. 7 and Table 2). In addition, apelin levels were negatively associated with pregnenolone and DOC concentrations, significantly greater in Control women (Fig. 7). Similarly, visfatin follicular concentration, which was higher in Control group, correlated negatively with that of Δ4-androstenedione and testosterone, which, by contrast, were predominant in PCOS group (Fig. 7). No significant correlation was found with respect to adiponectin and vaspin levels.

**Table 2:** Simple linear regression analysis between androgens and adipokines concentrations in follicular fluid of study women

	Resistin	Omentin	Chemerin	Apelin
**17OH-Pregnenolone**	0.038	0.053	0.054	0.042
**DHEA**	0.069	0.112	0.112	0.149
** Δ4-androstenedione**	0.048	0.066	0.094	0.142
**Testosterone**	0.037	0.088	0.073	0.101

Data are expressed as r^2^ (simple linear regression). *Note*: *DHEA* dehydroepiandrosterone; *DOC* deoxycorticosterone

## Discussion

In our study, we demonstrated that PCOS is associated with an intraovarian hyperandrogenic state characterised by a selective increase in androgens synthesised by TC, accompanied by a parallel decrease in androgens originating from the adrenals. To our knowledge, it is one of the first studies characterising the androgen profile in FF of PCOS women, and our results further strengthen the hypothesis that hyperandrogenism plays a key role in the pathogenesis of this complex endocrinopathy.

The presence of clinical and/or biological signs of hyperandrogenism is one of the major criteria required to establish PCOS diagnosis [1] and, despite some conflicting advice [25], for several years now androgen excess has been recognised as an essential factor in the development of most of the reproductive and metabolic alterations characterising this syndrome [7]. Although an adrenal origin can be identified in 10 to 30% of PCOS patients [5, 26], hyperandrogenism seems to result primarily from an intrinsic abnormality of ovarian TC, consisting of a dysregulation of the expression and/or activity of key steroidogenic enzymes [5, 27]. Gilling-Smith et al. first identified a hypersecretion of androstenedione, 17OHP and progesterone in culture medium of TC derived from polycystic ovaries, suggesting the possibility of a global overregulation of ovarian steroidogenesis mainly involving an increase in the 17-hydroxylase/17,20-lyase activity of cytochrome CYP17 [28]. These results were further confirmed in TC cultures from size-matched follicles of PCOS women and Controls, in which the authors also demonstrated a significant increase in the transcriptional expression of CYP17 and CYP11a, as well as in CYP17, 3βHSD and cytochrome P450 activity [6, 29]. Interestingly, the hypothesis of an intrinsic, possibly genetically determined, abnormality of TC at the origin of PCOS is further supported by genetic association studies. Indeed, the genes most frequently and significantly associated with this syndrome include several molecules involved in androgen metabolism, such as CYP11A, CYP21 [30] and, particularly, DENND1Av.2, a gene coding for a clathrin-binding protein that seems to play a key role in PCOS-associated hyperandrogenism by increasing CYP17A1 and CYP11A1 genes transcription [5, 31].

**Fig. 8:**
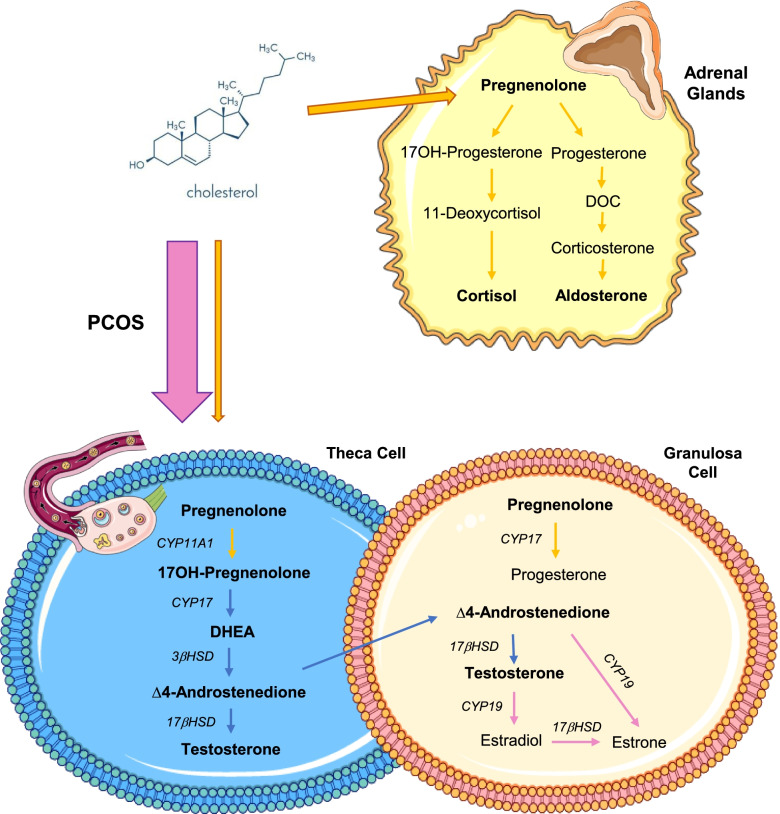
Overview of androgen synthesis pathways in women. Metabolic pathways of androgen synthesis in the adrenal glands and ovarian theca and granulosa cells. In PCOS (pink arrow), steroidogenesis seems to be diverted from adrenal to ovarian metabolic pathways due to CYP11A1, CYP17 and 3𝛽HSD hyperactivity in theca cells, resulting in increased intrafollicular levels of 17OH-pregnenolone, DHEA, ∆4-androstenedione and testosterone. PCOS = Polycystic Ovary Syndrome; DHEA = dehydroepiandrosterone; DOC = deoxycorticosterone

In our study, PCOS patients had significantly higher intrafollicular concentrations of 17OH-pregnenolone, DHEA, Δ4-androstenedione and testosterone than Controls and ECHO women. Particularly, at intrafollicular level, we showed a significant increase in 17OH-pregnenolone, that, according to Nelson et al. [29], could be explained by the predominant activity of CYP17A1 on 3βHSD. Interestingly, these findings corresponded to a significant decrease in pregnenolone, 17OHP, DOC and 11-deoxycortisol levels, raising the possibility that in PCOS, steroidogenesis is globally diverted from adrenal to ovarian metabolic pathways (Fig. 8). In agreement with several studies, the biochemical alteration typically characterising PCOS women consists of an exaggerated response of plasma 17OHP to treatment by hCG [4] or GnRH-agonists [32]. The fact that we found significantly lower levels of 17OHP in the FF of PCOS patients does not exclude the possibility that its plasma concentration was increased. Indeed, 17OHP is a central molecule in the adrenal synthesis of mineralocorticoids and glucocorticoids, which could be promoted in response to ovarian androgen overproduction.

The increase in plasma AMH and LH is one of the biological features typically, though variably, found in PCOS women [22]. Indeed, both hormones have been demonstrated to be tightly involved in the pathogenesis of the ovarian disorders characterising this endocrinopathy [33]. In agreement with this data, in our cohort, plasma AMH and LH concentrations were significantly higher in PCOS than in the other two groups and, interestingly, positively correlated with intrafollicular levels of 17OH-pregnenolone, DHEA, Δ4-androstenedione and testosterone.

AMH is nowadays recognised as the main hormonal regulator of ovarian follicular development by concurrently stimulating preantral follicles growth and inhibiting antral follicles maturation [22]. Its plasma levels are therefore considered to reflect the extent of ovarian reserve, as well as intrafollicular androgenic state as androgens promote the early stages of folliculogenesis [5]. According to this hypothesis, in a PCOS cohort, Rosenfield and Ehrmann found that plasma AMH concentration was independently associated with the presence of PCOM and intraovarian hyperandrogenism [5]. The positive correlations found in our study between plasma AMH and TC-derived androgens, as well as between follicle count and testosteronemia, strengthen the association between PCOS and intrafollicular hyperandrogenism and, at the same time, corroborate literature data supporting the pathogenic role of androgen excess in the development of PCOM [34]. Indeed, in women, any condition of hyperandrogenism, whether of endogenous (e.g., congenital adrenal hyperplasia) or exogenous (e.g., female-to-male transsexualism) origin, induces in the ovaries the anatomical and histopathological features characteristic of PCOS women [34]. Similarly, in rhesus monkey and sheep models, adult females exposed in utero to high doses of testosterone show large ovaries containing many small follicles, increased LH levels and dysovulation [35].

LH hypersecretion resulting from dysregulation of the hypothalamic-pituitary axis, as besides being at the origin of the oligo-anovulation characteristic of PCOS, contributes in a variable way depending on the patient, to the development of hyperandrogenism by stimulating TC steroidogenesis [5]. Androgen excess, in turn, appears to disrupt the negative feedback of oestradiol and progesterone on pituitary, resulting in an increase in frequency and amplitude of LH secretory pulses [36]. Interestingly, in our cohort, intrafollicular levels of 17OH-pregnenolone, DHEA, Δ4-androstenedione and testosterone positively correlated with plasma LH concentration and cycles duration, a result that further supports the existence of a close association between intraovarian hyperandrogenism and LH, possibly critical in the development of ovulatory disorders typical of PCOS.

The demonstration of a significant association between androgens and most of the adipocytokines of interest suggests the existence of a tight relationship between these two hormonal systems and further confirms the possible involvement of adipocytokines in ovarian physiopathology. In a previous study [23], we already analysed adipokines expression in the FF of PCOS patients and found high levels of apelin, omentin, chemerin and resistin. Conversely, visfatin concentration was significantly lower in the obese PCOS subgroup. These findings are confirmed by the results of the present study obtained in a larger cohort of patients and are further strengthened by the associations found with the intrafollicular levels of the androgens predominant in PCOS women, i.e. positive correlation with apelin, omentin, chemerin and resistin concentrations, and negative correlation with visfatin. On the other hand, according to our previous finding [24], vaspin does not seem to be directly implicated in PCOS pathogenesis, particularly as regards intraovarian hyperandrogenism. Indeed, although an increase in vaspin protein expression and secretion was reported in TC-granulosa cells (GC) cocultures after testosterone stimulation [37], literature generally rules out any significant association between this adipokine and androgens in PCOS women [38].

Evidence concerning androgens-adipokines relationships, particularly at ovarian level, is currently scarce and often inconclusive. Nevertheless, chemerin plasma concentration has been reported to be higher in hyperandrogenic PCOS patients than in the normo-androgenic ones and to correlate positively with testosterone levels [39]. Additionally, CMKLR1 gene deletion protected DHT-treated rats from the negative effects of androgen treatment on progesterone secretion, cycles regularity and ovulation [39]. Considering these results and the fact that, in vitro, testosterone stimulation of human GC resulted in an overexpression of both chemerin and its receptors [40], Lima et al. suggested that hyperandrogenism might induce an increase in chemerin ovarian levels, which, in turn, might act as a chemoattractant for circulating CMKLR1+ monocytes [41]. Supporting this hypothesis, in DHT-treated rats, hyperchemerinemia was associated with increased apoptosis of GC [42], and the number of the oocytes and embryos obtained from women with high intrafollicular chemerin concentrations was significantly reduced [40]. According to this data, chemerin could therefore be one of the possible mediators through which hyperandrogenism induces the perturbations in folliculogenesis and the alterations in oocyte maturation at the origin of PCOS [42].

Similarly, adipocyte expression of resistin has been shown to be higher in PCOS women with hyperandrogenism [43], and its plasma concentration positively correlated with testosteronemia in PCOS patients but not in Controls, evoking the possibility that polycystic ovary TC are more sensitive to the action of resistin, which, by interacting with insulin, could contribute to androgen hyperproduction [44].

An analogous mechanism has been proposed for omentin. Indeed, in human GC, omentin has been demonstrated to facilitate IGF-1 signalling by increasing cell sensitivity to insulin [45]. Since IGF-1, acting in synergy with LH, stimulates androgen production by TC [45], omentin could be involved in the development of hyperandrogenism, particularly in obese subjects. However, it is noteworthy that, according to several studies, in PCOS patients, adipocyte synthesis of omentin is decreased in conditions of hyperandrogenism and its plasma concentration negatively correlates with free testosterone levels [46, 47]. These results, although somewhat controversial [48], contrast with our observations, suggesting that the relationships between omentin and androgens are much more complex and deserve further investigation.

Likewise, literature on visfatin does not agree with our results. Indeed, visfatin has been proposed to stimulate androgen synthesis by TC through its insulin-like action [49]. Furthermore, in two different cohorts of PCOS patients, hyperandrogenic women [49] and hirsute adolescents [50] had significantly higher visfatin plasma levels than normo-androgenic and non-hirsute subjects, and a positive correlation between circulating visfatin and several markers of hyperandrogenism was repeatedly found [49–51]. Considering that these results refer to visfatin plasma expression, the discrepancy with our findings might indicate that this adipocytokine is differently regulated at systemic and ovarian level. However, consistent with our previous data [23], its implication in PCOS pathogenesis seems to be limited.

As regards apelin, the relationship between this adipocytokine and PCOS hyperandrogenism has been widely studied and appears mostly indirect. Indeed, apelin has been reported to be involved in hormonal regulation of ovarian function, particularly in folliculogenesis, via its action on the arcuate, supraoptic and paraventricular hypothalamic nuclei resulting in suppression of FSH, LH and prolactin secretion [52]. In agreement with this data, a negative correlation between plasma concentrations of apelin and LH was repeatedly described [53]. Our results could therefore suggest that apelin contributes to steering steroidogenesis towards preferential androgen synthesis through the perturbations induced at HPG axis level, which, moreover, would play a key role in arresting follicular development at the origin of PCOM [23].

In our study, we characterised for the first time the expression profile of androgens in FF, revealing intraovarian hyperandrogenism in PCOS patients. Although this result is very significant by itself considering that most of the women had no signs of systemic hyperandrogenism, it would have been even more relevant if we could have had the testosteronemia data for all included subjects, particularly those obtained at the time of FF collection. Indeed, this would have made it possible to compare androgen levels in plasma and FF, and to analyse their relationships with adipocytokines and features of interest. A new study already underway in our laboratory will enable us to obtain a blood sample on the same day as the oocyte punction, thus overcoming this limitation and possibly highlighting other significant aspects of ovarian physiopathology. In analogy to what has been done previously in human GC [24, 45, 54, 55], it would be noteworthy also to evaluate the expression of the main steroidogenic enzymes in TC from PCOS, ECHO and Control women stimulated with different adipocytokines. This could allow us to validate or refute our hypotheses about AMH and LH involvement in ovarian steroidogenesis and, above all, about the interactions between androgens and adipocytokines in PCOS pathogenesis.

## Conclusions

In conclusion, we demonstrated for the first time that PCOS is characterised by a selective increase in intrafollicular levels of the androgens synthesised by TC, strengthening the hypothesis that ovarian hyperandrogenism plays a central role in the pathogenesis of this complex syndrome. Moreover, the demonstration of a significant association between follicular androgens and most of the adipokines of interest, including apelin, chemerin, resistin and omentin, confirms the existence of a close relationship between these two hormonal systems, that appear deeply involved in ovarian physiology and, more so, in PCOS physiopathology.

## Data Availability

The datasets used and/or analysed during the current study are available from the corresponding author on reasonable request.

## Abbreviations

AMH: Anti-Mullerian Hormone
BMI: Body Mass Index
CMKLR1: Chemerin Chemokine-Like Receptor 1
CYP17A1: Cytochrome P450 family 17 subfamily A member 1
CYP11A1: Cytochrome P450 family 11 subfamily A member 1
CYP17: Cytochrome P450 family 17 subfamily A member 1
DHEA: Dehydroepiandrosterone
DOC: Deoxycorticosterone
FF: Follicular Fluid
FSH: Follicle Stimulating Hormone
GC: Granulosa Cells
IGF-1: Insulin-like Growth Factor I
IVF: In vitro Fertilisation
LH: Luteinising Hormone
PCOM: Polycystic Ovary Morphology
PCOS: Polycystic Ovary Syndrome
TC: Theca Cells
